# Role of Compartmentalization on HiF-1α Degradation Dynamics during Changing Oxygen Conditions: A Computational Approach

**DOI:** 10.1371/journal.pone.0110495

**Published:** 2014-10-22

**Authors:** Baptiste Bedessem, Angélique Stéphanou

**Affiliations:** UJF-Grenoble 1, CNRS, Laboratory TIMC-IMAG/DyCTIM, UMR 5525, La Tronche, France; Georgia Regents University, Medical College of Georgia, United States of America

## Abstract

HiF-1*α* is the central protein driving the cellular response to hypoxia. Its accumulation in cancer cells is linked to the appearance of chemoresistant and aggressive tumor phenotypes. As a consequence, understanding the regulation of HiF-1*α* dynamics is a major issue to design new anti-cancer therapies. In this paper, we propose a model of the hypoxia pathway, involving HiF-1*α* and its inhibitor pVHL. Based on data from the literature, we made the hypothesis that the regulation of HiF-1*α* involves two compartments (nucleus and cytoplasm) and a constitutive shuttle of the pVHL protein between them. We first show that this model captures correctly the main features of HiF-1*α* dynamics, including the bi-exponential degradation profile in normoxia, the kinetics of induction in hypoxia, and the switch-like accumulation. Second, we simulated the effects of a hypoxia/reoxygenation event, and show that it generates a strong instability of HiF-1*α*. The protein concentration rapidly increases 3 hours after the reoxygenation, and exhibits an oscillating pattern. This effect vanishes if we do not consider compartmentalization of HiF-1*α*. This result can explain various counter-intuitive observations about the specific molecular and cellular response to the reoxygenation process. Third, we simulated the HiF-1*α* dynamics in the tumor case. We considered different types of mutations associated with tumorigenesis, and we compared their consequences on HiF-1*α* dynamics. Then, we tested different therapeutics strategies. We show that a therapeutic decrease of HiF-1*α* nuclear level is not always correlated with an attenuation of reoxygenation-induced instabilities. Thus, it appears that the design of anti-HiF-1*α* therapies have to take into account these two aspects to maximize their efficiency.

## Introduction

The HiF-1 factor is the central protein involved in the intracellular signaling pathway of hypoxia [1]. This transcription factor, which enhances the expression of numerous genes, directs the very complex cellular response to hypoxia. HiF-1 is an heterodimeric protein, constituted of two sub-units: HiF-1*β*, which is constitutively expressed, and HiF-1*α*. The concentration of HiF-1*α* is maintained at low levels in most cells under normoxic conditions. Whereas its synthesis does not depend on oxygen level [2], its degradation is regulated by the hypoxic state of the cell [3]. In normoxia, enzymes called prolyl-hydroxylase (PHD) are active and convert HiF-1*α* into an hydroxylated form. This form of the protein has a great affinity for an ubiquitination complex linked to the pVHL protein [4]. As a consequence, it is rapidly degraded by the proteasom pathway [3]. During a hypoxic event, the PHDs are inactive, and pVHL cannot bind to HiF-1*α*, which is not addressed to the proteasom anymore. Therefore, the transcription factor accumulates and drives the synthesis of numerous genes, involved in cell cycle arrest and apoptosis [5], or, on the contrary, in proliferation [6], [7] and survival [8].

It is well known that HiF-1*α* accumulates in most of tumor cells [9]–[11]. This accumulation is first due to the chronic hypoxia underwent by tumor cells [12], [13]. Second, many mutations can deregulate HiF-1*α* level, by impairing the HiF-1*α*/pVHL interaction [14]–[16], or its genetic expression [17]–[19]. Because of the anti-apoptotic effects of HiF-1, this overexpression plays a crucial role in tumor growth [20]. Indeed, it was shown that hypoxia generates chemoresistant cells, and then participates to the emergence of very aggressive tumor phenotypes [13], [21]. As a consequence, the understanding of the mechanisms driving the accumulation and degradation of HiF-1*α* is crucial to identify new targets for anti-cancer therapies [22].

The features of HiF-1*α* accumulation under hypoxia are well known. HiF-1*α* rapidly increases after the beginning of a hypoxic event, and reaches a maximum after a certain time depending on the tissue (1 h–5 h) [23]. Then, it gradually decreases down to an equilibrium level [24], [25]. This equilibrium level depends on the intensity of hypoxia, following an exponential law determined experimentally by Jiang et al. (1996) [26]. As a consequence, HiF-1*α* begins to significantly accumulate when oxygen pressure passes through a threshold (about 1% O_2_). This switch-like accumulation of HiF-1*α* is considered as an important feature of the response to hypoxia [27]. If hypoxia is very intense (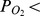
0.1%) the apoptotic pathways modify the equilibrium value of HiF-1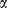 and promote apoptosis [8], [28]. In this work, we do not consider the relationships between the hypoxic and apoptotic pathways. At the molecular scale, the pathways regulating HiF-1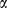 induction and degradation are complex and not perfectly known. The formation of the pVHL/HiF-1*α* complex is essential to address the transcription factor to the proteasom [29], [30]. The hydroxylation of a proline residue of a specific domain of HiF-1*α*, called the ODD domain, makes possible the formation of the complex [31]. The chemistry of the pVHL/HiF-1*α* complex was studied *in vitro*. It was notably shown that during the passage from hypoxia to normoxia, the affinity of pVHL for HiF-1*α* is multiplied by at least three orders of magnitude [32], [33].

In normoxia, when protein synthesis is blocked (by the cycloheximide), the degradation of HiF-1*α* follows a bi-exponential law experimentally identified by Moroz et al. (2009) [25]. The authors showed that in a line of normal cells (NIH3T3), the two half-times of the HiF-1*α* degradation law are 6
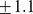
min and 217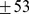 min. They made the hypothesis that the slow clearance of HiF-1*α* was due to its nuclear degradation, whereas the rapid one reflected the cytoplasmic degradation of HiF-1*α*. This idea is interesting because it contributes to the debate concerning the sub-cellular localization of the protein. A general description is given as follows: in normoxia, HiF-1*α* level is very low and when it is detectable, the protein is mainly cytoplasmic. When hypoxia is important, HiF-1*α* rapidly accumulates in the nucleus [34]–[36]. Recently, some experimental works have shown that there is a dynamics of export/import of HiF-1 from the nucleus to the cytoplasm, which is crucial to regulate its degradation. After its synthesis in the cytoplasm, HiF-1*α* is constitutively imported into the nucleus [25], [37]. On the other hand, the pVHL protein is involved in a permanent shuttle between the nucleus and the cytoplasm [38], [39]. In normoxia, as the pVHL/HiF-1*α* complex can form, HiF-1*α* is exported to the cytoplasm, where it is degraded, as hypothesized by Groulx et al. (2002) [40]. The dynamics of the HiF-1*α*/pVHL shuttle may be highly variable. Zheng et al. (2006) [41] showed that for highly proliferating cells, HiF-1*α* exclusively accumulates in the nucleus during hypoxia, and its degradation mainly takes place in the cytoplasm during normoxia. This result suggests that in this type of cells, the export rate is much more important than the import rate. This agrees with the result that pVHL is mainly a cytoplasmic protein [42]. However, in differentiated, non-proliferating cells, HiF-1*α* degradation takes place equally in the cytoplasm and in the nucleus [41]. These results are coherent with the observation that pVHL is predominantly in the cytoplasm of confluent (non-proliferating) cells, and in the nucleus of sparse cells [38]. We can thus suppose than the rate of pVHL export is highly variable depending on the cell type or state: high for quiescent/slowly proliferative cells, and low for proliferative cells. Besides, Zheng et al. (2006) [41] supposed that the HiF-1*α* nuclear degradation observed is due to the activity of a nuclear proteasom. This hypothesis can be linked to that formulated by Moroz et al. (2009) [25]. However, it is still in debate, and the importance of the nucleo-cytoplasmic shuttle of HiF-1*α* is not well established. Notably, an important question is to understand why HiF-1*α* rapidly accumulates in the nucleus (switch-like behavior) when the oxygen pressure passes through a threshold. How can the nuclear/cytoplasmic shuttle explain this typical feature of HiF-1*α* dynamics?

The effects of intermittent hypoxia on HiF-1*α* accumulation are well studied. The studies by Martinive et al. (2006) [43] and Martinive et al. (2009) [44] demonstrate that HiF-1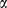 accumulates during hypoxia/reoxygenation cycles. This result was confirmed by Yuan et al. (2008) [45] and Dewhirst et al. (2007) [46]. Besides, the effects of a reoxygenation event on the expression of hypoxia related genes are well known. Various authors reported that reoxygenation stimulates the expression of HiF-1-dependent genes. Conde et al. (2012) [47] demonstrate that classical genes related to HiF-1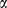 activity, such as VEGF, PHD, EPO, are over-expressed during reoxygenation. Hsieh et al. (2009) [48] obtained a similar result by using reporter genes. The authors interpreted this result by an increase of HiF-1 activity during reoxygenation. Luo et al. (2010) [49] demonstrated that the protein MICA, present in cell overexpressing HiF-1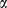, is strongly expressed during reoxygenation. They showed that this expression was linked to HiF-1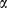. Reoxygenation is also known to induce apoptosis [50], [51]. Wang et al. (2012) [52] showed that this reoxygenation-induced apoptosis was due to HiF-1*α*. These observations lead to formulate the hypothesis that there is a specific response of HiF-1*α* level or genetic activity during reoxygenation, different from that of normoxia. The study by Conde et al. (2012) [47], confirms this idea, by showing an instability of HiF-1 level during 6 hours after reoxygenation. The level rapidly decreases after 15 min, and increases to reach a maximum at 3 h (level close to the hypoxic level). Then, HiF-1 level decreases. The authors bring elements showing that this up-regulation of HiF-1 was maybe due to the AKm/Tor pathway.

Thus, there is clearly a tendency for HiF-1 level and genetic activity to become unstable after reoxygenation. The causes of such a tendency are not perfectly known.

Many models of HiF-1 regulation pathways were developed over these last ten years, to explain the main features of HiF-1*α* accumulation during hypoxia [27], or its effects on cell cycle [53]. Since the work by Kohn et al. (2004) [54], these models aim to simulate the principal features of HiF-1 response to hypoxia, mainly by taking into account the oxygen-dependent action of the PHDs. In various works, the HiF-1*α* dynamics is explained by considering feedback phenomena, such as the HiF-1-dependent synthesis of PHDs (Kohn et al. (2004) [54]), or the inhibition of PHDs by succinate (Qutub et al. (2007) [55]). The model by NGuyen et al. (2013) [56] includes the influence of FIH on transcriptional activity of HiF-1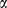, and also includes a compartmentalization aspect. However, it does not consider the pVHL shuttle, and does not study the effects of variations of the export/import dynamics of the PDHs/FIH on HiF-1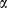 regulation. Yet, it appears that this dynamics is complex and depends on the cell type and on the cell state [57]–[59]. Some authors have made a theoretical analysis of the system of ODEs initially given by Kohn et al. (2004) [54]. Yu et al.(2007) [28] made an Extreme Pathway Analysis, and Heiner et al. (2010) [60] a structural analysis. The aim of this approach is to identify the major pathways responsible for the switch-like behavior. It is a way to extract the network corresponding to the complex response to hypoxia.

In this study, we decided to consider that a possible core pathway of HiF-1 regulation was its pVHL-dependent degradation, driven by an oxygen-dependent affinity of pVHL for HiF-1, and the HiF-1*α* feedback on pVHL synthesis. First, it is well known that HiF-1*α* stability is driven by its affinity with pVHL [4]. This affinity varies with PHDs' activity, which depends on the oxygen level, as assumed in the previous models [55]. Second, there is no model taking into account the interesting feedback of HiF-1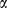 on pVHL mRNA synthesis. The existence of this feedback is well accepted in the literature, as noticed in the recent review by Sermeus et al. (2011) [8]. Two main arguments can be used to justify this hypothesis. First, the promotor of the pVHL gene exhibits a putative site for HiF-1 fixation, as noticed by Blagosklonny et al. (2001) [61], based on previous results of Renbaum et al. (1996) [62]. The presence of this HRE (HiF-1 responsive element) was confirmed by Krausen et al. (2005). Besides, the authors show a direct binding of HiF-1 on the pVHL gene promotor. Second, Karhausen et al. (2005) demonstrate that pVHL mRNA and pVHL protein are upregulated during hypoxia [63]. This result was not contradicted by more recent studies [8]. As a consequence, we considered that the pVHL-HiF-1 feedback is a relevant hypothesis to study. That is why we consider it, for the first time, in a model of HiF-1*α* regulation dynamics.

Our model simulates the HiF-1*α* dynamics by taking into account, for the first time, the nucleo-cytoplasmic shuttle. We consider two compartments (nucleus and cytoplasm), where HiF-1*α* is degraded at different rates. The action of PHDs is not considered. However, we consider a parameter describing the oxygen-dependent affinity of pVHL for HiF-1*α*. The value of this parameter decreases with oxygen pressure. Thus, it reflects the activity of PHDs, following a well-known model: when the activity is high (normoxia), HiF-1 is hydroxylated and its affinity for pVHL is high. When the activity is low (hypoxia), HiF-1 affinity for pVHL decreases. With this simple model, we can explain the bi-exponential degradation law of HiF-1*α*, and confirm the importance of considering two compartments for the degradation. We also well describe the kinetics of HiF-1*α* under hypoxia, and its switch-like nuclear accumulation. We can also provide a simple explanation to the counter-intuitive increase of the hypoxic response after reoxygenation. We show that a hypoxia/reoxygenation cycle induces an oscillatory pattern of HiF-1*α* level which can explain the specific cellular effects of reoxygenation, notably apoptosis and enhanced expression of hypoxia-regulated genes. This instability is directly linked to the compartmentalization of HiF-1*α* degradation dynamics. Finally, our model can be used to model the effects on HiF-1*α* levels of different kinds of cancerous mutations, and to test the potential efficiency of different therapeutic strategies.

## Model

### Biological description

**Figure 1:**
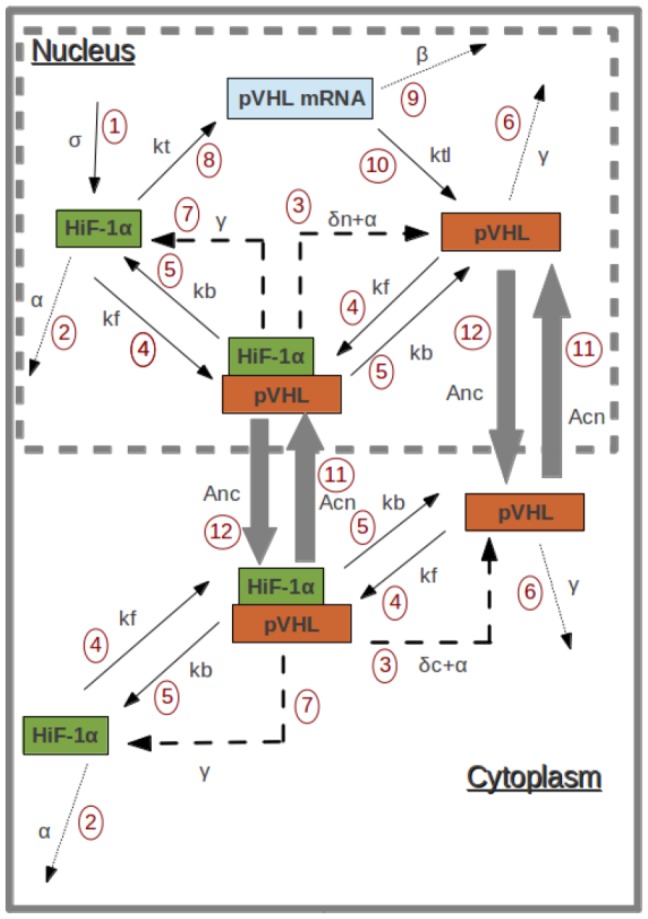
Molecular network of HiF-1*α* regulation. Sketch of the molecular network considered in the model.

On the basis of data from the literature about the dynamics of HiF-1*α* degradation and nucleo-cytoplasmic exchanges, we constructed a mathematical model comprising seven ordinary differential equations (ODEs). The variables used in the model are presented in Table 1. Because of the similarities between the pVHL/HiF-1*α* system and the p53/MdM2 system [61], we used the same formulation as Hunziker et al. (2010) [64] to write our ODEs system. In each compartment, we consider that the system is driven by the mass-action law which characterizes the pVHL/HiF-1*α* complex assembly/disassembly dynamics. The formation of the complex leads, in both compartments, to HiF-1*α* degradation. As HiF-1*α* is constitutively imported into the nucleus after its synthesis, we hypothesized that it appears in the nucleus, with a synthesis rate belonging to the parameters of the system. The feedback of HiF-1 on pVHL synthesis is modeled by a HiF-1-dependent rate of pVHL mRNA synthesis. We chose the same expression for this dependency as Hunziker et al. (2010) [64]. We then consider, because of the presence of a nuclear-localization signal in pVHL protein, that the protein is constitutively imported into the nucleus after its synthesis [65]. Its accumulation in the cytoplasm is due to the nucleo-cytoplasmic shuttle. This shuttle is described with an import and export rate of pVHL, which ensures the coupling between the cytoplasm and the nucleus dynamics. Figure 1 represents the different reactions modeled by the ODEs system. Table 2 gives the meanings of each reaction represented in figure 1. Table 3 gives the link between the parameters and these reactions.

**Table 1:** Variables of the model.

Model variable	Associated molecule
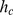	Cytoplasmic HiF-1*α*
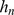	Nuclear HiF-1*α*
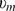	pVHL mRNA
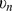	Nuclear pVHL
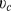	Cytoplasmic pVHL
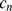	Nuclear pVHL/HiF-1*α* complex
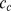	Cytoplasmic pVHL/HiF-1*α* complex

Biological molecules considered in the model, and their mathematical symbol.

**Table 2:** Reactions of the model.

Number on the figure 1	Reaction
1	Synthesis of HiF-1*α*
2	pVHL-independent degradation of HiF-1*α*
3	Release of pVHL by pVHL-dependent and pVHL-independent degradation of HiF-1*α*
4	Formation of the pVHL/HiF-1*α* complex
5	Dissociation of the pVHL/HiF-1*α* complex
6	pVHL natural degradation
7	Release of HiF-1*α* by pVHL degradation
8	HiF-1-dependent synthesis of pVHL mRNA
9	pVHL mRNA degradation
10	Synthesis of pVHL
11	pVHL-import rate into the nucleus
12	pVHL-export rate into the cytoplasm

Meaning of the reactions of the model. The numbers refer to the figure 1.

**Table 3:** Parameters of the model.

Parameter	Description	Reactions of the figure 1
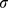	Synthesis rate of HiF-1	1
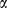	pVHL-independent degradation of HiF-1	2,3
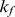	pVHL/HiF-1 complex formation rate	4
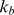	pVHL/HiF-1 complex dissociation rate	5
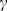	Degradation rate of pVHL	6,7
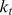	Synthesis rate of pVHL mRNA	8
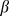	Degradation rate of pVHL mRNA	9
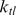	Synthesis rate of pVHL protein	10
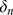	pVHL-dependent degradation rate of HiF-1 in the nucleus	3
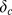	VHL-dependent degradation rate of HiF-1 in the cytoplasm	3
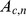	Import rate of pVHL/HiF-1 complex into the nucleus	11
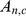	Export rate of pVHL/HiF-1 complex into the cytoplasm	12

Definition of the parameters used in the model. Each parameter quantifies the speed of a reaction represented in figure 1.

### ODEs system

We present here the 7 ordinary differential equations used in our model. We consider the following variables that account for the concentrations of the chemical species: 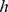 (total HiF-1), 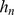 (nuclear HiF-1), 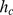 (cytoplasmic HiF-1), 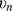 (nuclear pVHL), 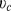 (cytoplasmic pVHL), 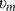 (pVHL mRNA), 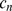 (nuclear (pVHL/HiF-1*α* complex), 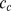 (cytoplasmic pVHL/HiF-1*α* complex). The meaning and the value of the parameters are summarized in Tables 3 and 4. The equations were solved using a Runge-Kutta method, implemented in Matlab.

**Table 4:** Values of the parameters for normal, slowly proliferating cells.

Parameter	Default value	Range of variations	Reference
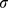	1000 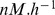	[200 8000]	Moroz et al. (2009)
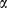	0.27 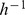		Moroz et al. (2009)
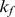	1000 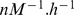	[0 1000]	Moroz et al. (2009)
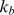	7200 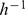		Hunziker et al. (2010)
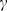	0.8 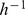		Yang et al. (2013)
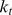	0.001 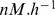		Moroz et al. (2009)
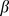	0.6 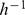		Hunziker et al. (2010)
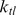	1.4 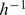		Hunziker et al. (2010)
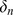	1 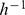		Moroz et al. (2009)
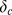	7 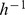		Moroz et al. (2009)
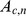	10 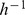		Moroz et al. (2009)
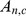	1000 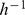	[10 1000]	Moroz et al. (2009)

Default values of the parameters, for a normal, non-proliferating cell, and range of variations of the parameters used in the simulations. The default values were fixed using previous models (Hunziker et al. (2010)), or experimental data (Moroz et al. (2009), Yang et al. (2013)).



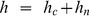
(1)This first equation accounts for the total conservation of HiF-1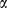 which is distributed between the cytoplasm and nucleus of the cell.
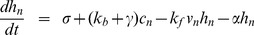
(2)


The nuclear HiF-1 is synthesized with rate 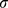, spontaneously degraded with rate 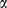, and is engaged into a chemical equilibrium with the pVHL/HiF complex. 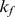 and 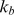 are the assembly/disassembly rates of the complex. The degradation of pVHL in the nuclear complex liberates HiF-1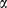 with rate 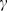


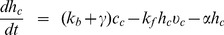
(3)


Equation similar to that for the nuclear HiF-1, but without the synthesis term. Indeed, HiF-1*α* is constitutively imported into the nucleus after its synthesis [25], [37]. We thus consider that it appears in the nucleus, and that its presence in the cytoplasm is due to the nucleo/cytoplasmic shuttle
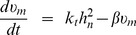
(4)


Equation for pVHL mRNA. Its synthesis is promoted by HiF-1 with rate 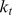, and it is degraded with rate *β*. We chose the same law as Hunziker et al. (2010) [64] for the MdM2/p53 system.
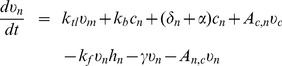
(5)


Equation for the nuclear pVHL. It is synthesized from mRNA with rate 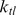, and naturally degraded with rate 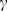. It is engaged in a chemical equilibrium with the pVHL/HiF complex. 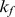 and 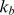 are the assembly/disassembly rates of the complex. This complex leads to the nuclear proteasomal degradation of HiF-1 with rate 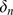 which liberates pVHL. The spontaneous degradation of HiF-1*α* also liberates pVHL with rate *α*.
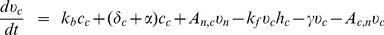
(6)


Equation for the cytoplasmic pVHL. Similar as that for the nuclear pVHL, but without the synthesis term. Indeed, we made the hypothesis that pVHL is constitutively imported into the nucleus after its synthesis. We thus consider that it appears in the nucleus, and that its presence in the cytoplasm is due to the nucleo/cytoplasmic shuttle
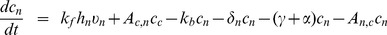
(7)


Equation for the nuclear pVHL/HiF-1 complex. It is assembled with rate 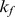, and disassembled with rate 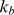. The spontaneous degradations of pVHL and HiF-1*α*, and the pVHL-induced HiF-1 degradation decreases its concentration with rates 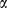, 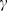 and 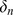 respectively. The nuclear complex is exported to the cytoplasm with rate 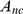, and the cytoplasmic complex is imported with rate 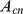.
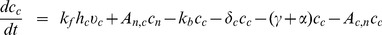
(8)


Equation for the cytoplasmic complex. Similar as that for nuclear complex.

The method used to solve this system and run the simulations are presented as Material S1 & S2. Material S1 describes the methods we used to find the stationary state of the system, and to simulate hypoxia or reoxygenation events. Material S2 gives the Matlab code used to obtain the main results of the paper.

### Determination of some parameters

The value of the natural degradation rate of HiF-1*α* (*α*) can be determined by using data from Moroz et al. (2009) [25]. The authors showed that in ODD-mutant cells, the half-time of HiF-1*α* degradation was 160 min (2.7 h). Since the ODD-mutant cells cannot form HiF-1*α*/complex, this experimental time can easily be used to estimate the value of the 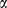 rate. We fix 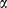 = 0.27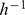. We also have a value for the pVHL degradation rate (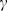), from Yang et al. (2013) [66]. The authors measured a half-life of 3.8 hours for pVHL, which corresponds to a parameter 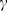 = 0.2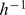. The other parameters are not precisely known. To reduce the number of degrees of freedom of the system, we used the analysis of Hunziker et al. (2010) [64] on the modeling of the p53/MdM2 dynamics. The authors fixed the value of the MdM2 mRNA natural degradation rate 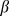 to 0.6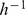, corresponding to a half-time of about 1 hour. Besides, they noticed that this value was not important to determine the dynamics of the system, and we verified that it was also the case in our model. Thus, we chose to fix 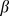 = 0.6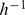. In a similar way, the translation rate 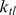 of MdM2 was fixed in order to ensure small values of free p53 at the equilibrium (about 100 nM) [64]. As the experimental observations made on HiF-1*α* are coherent with this idea (undetectable levels of HiF-1*α* in normoxia), we fix 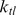 = 1.4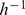. We also verified that its variations do not influence the dynamics of the system. The other parameters (HiF-1*α* synthesis rate 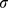, formation and disassembly of pVHL/HiF-1*α* 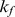 and 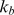, pVHL-dependent degradation rates of HiF-1*α* 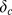 and 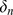, pVHL export and import rates 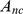 and 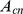, HiF-1*α* dependent induction rate of pVHL 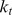) are the degrees of freedom of our system. As the pVHL/HiF-1*α* complex level depends on the 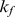/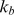 ratio, we can consider that only one of this two parameters drives the dynamics of the system. Thus, we chose to fix 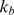 (disassembly rate of the pVHL/HiF-1*α* complex), and to consider only the variations of 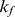 (formation rate of the complex, depending on the oxygen level). We chose the default value given by Hunziker et al. (2010) for the p53/MdM2 system: 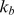 = 7200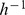. To give a default value to the remaining undetermined parameters, we then used the bi-exponential HiF-1 degradation law given by Moroz et al. [25].

## Results

### Simulation of HiF-1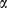 degradation in normoxia

**Figure 2:**
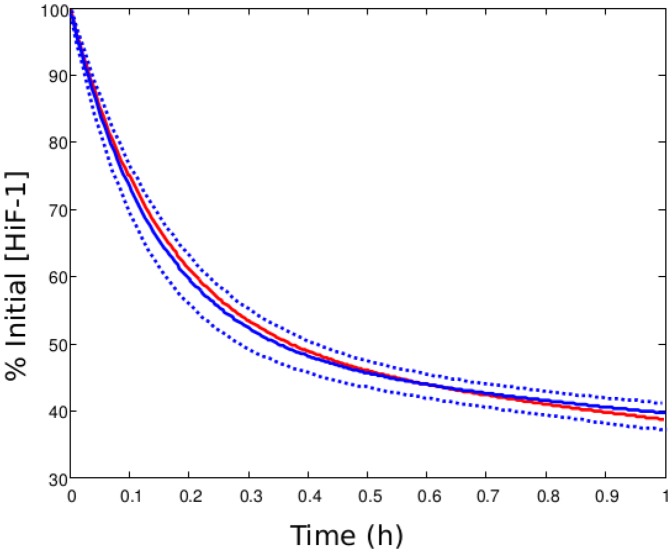
Experimental and simulated HiF-1*α* degradation curves. Blue: experimental degradation curve determined by Moroz et al. (2009) [25] after the addition of cycloheximide. Solid line: mathematical bi-exponential law determined with experimental data. Dotted lines: uncertainty on the bi-exponential law. Red: simulated degradation curve. The total HiF-1*α* level (free and complexed, cytoplasmic and nuclear) is plotted as the percentage of its initial value.

We first consider the experimental situation described by Moroz et al. (2009) [25]. Cycloheximide is added to a culture of NIH3T3 cells (differentiated, slowly proliferating cells) in normoxia in order to block protein synthesis. Then, HiF-1*α* concentration is measured along time in order to construct its degradation curve. The authors determined a bi-exponential degradation law for HiF-1 degradation: [HiF-1](t) =  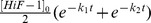 with [HiF-1](t) the level of [HiF-1] along time, [HiF-1]_0_ its initial level, and 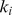 a characteristic parameter such as 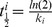. Searching for a bi-exponential law of degradation means searching the value of these two parameters. The experimental values of these two half-times are: 6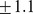 min and 217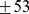 min. This experimental protocol corresponds, in our model, to the calculation of the stationary state of the system, and then to fix 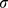 (HiF-1*α* synthesis rate) and 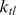 (pVHL mRNA translation rate) = 0. We then calculated the degradation curve, that is to say the evolution of total HiF-1 concentration along time. By using a least-square method, we searched for the better bi-exponential law fitting this curve, following the same approach as Moroz et al. (2009) [25]. We made this simulation for 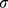 (HiF-1*α* synthesis rate), 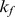 (complex formation rate), 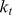 (HiF-1*α* induced pVHL synthesis rate), 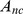, 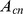 (pVHL export and import rates), and 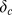, 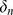 (cytoplasmic and nuclear pVHL-dependent HiF-1*α* degradation rates) varying in biologically relevant ranges. For each set of parameters tested, the bi-exponential law obtained was compared to the experimental data from Moroz et al. (2009) [25]. We could thus identify a set of parameters ensuring a very good fit between theoretical and experimental degradation laws. Figure 2 shows the comparison between the simulated and the experimental degradation curves, obtained with the parameters presented in Table 4. The default values of the parameters correspond to the values ensuring the best fit. We reproduced the rapid clearance, corresponding to cytoplasmic degradation, and the slow one, corresponding to nuclear degradation. These parameters have a good biological relevance. The synthesis rate of HiF-1*α* is the same as the one used by Hunziker et al. (2010) [64] for p53, and the value of the complex formation rate (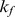) is in the range determined for the p53/MdM2 complex. This agrees with the idea of the symmetry between the p53/MdM2 and the HiF-1*α*/pVHL couple. The values of the pVHL-dependent HiF-1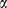 nuclear and cytoplasmic degradation rates (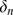 and 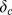) correspond to a bigger proteasomal activity in the cytoplasm than in the nucleus for normal cell, which agrees with experimental data [41]. Finally, the pVHL export rate (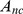) is much bigger than its import rate (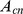), which corresponds to the experimentally observed massive export of pVHL out of the nucleus of non-proliferating cells [38], [42]. We notice that even if we have many undetermined parameters (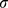, 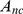, 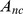, 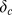, 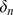, 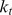, 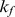), the bi-exponential curve is sufficient to constrain the parameters of the models to realistic values. Indeed, it gives the characteristic times of nuclear and cytoplasmic degradations, which impose the degradation rate in each compartment, the exchanges between them, and the HiF-1*α*/pVHL interactions.

### Simulation of HiF-1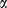 level in hypoxia

**Figure 3:**
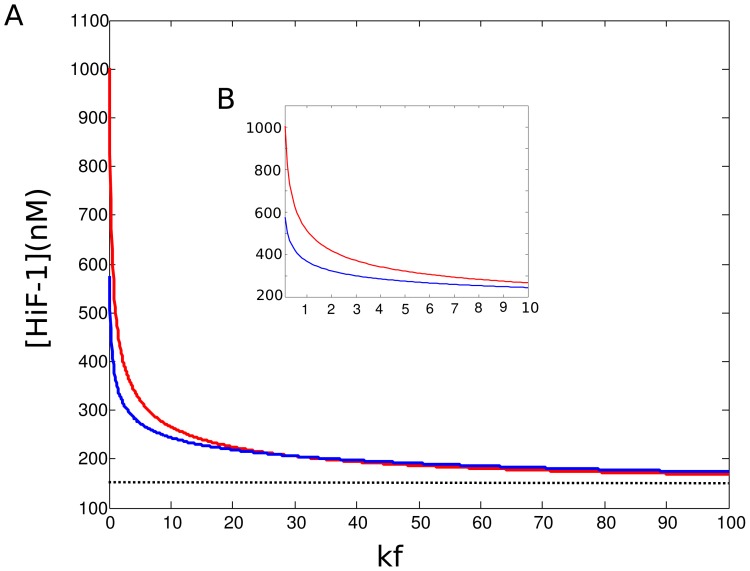
Accumulation of HiF-1*α* under hypoxia. Nuclear (red curve) and cytoplasmic (blue curve) equilibrium levels of HiF-1*α* are plotted as functions of the pVHL/HiF-1*α* complex formation rate (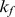). 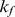 varies over a [1 100] range (A), and a [0.1 10] range (B).

**Figure 4:**
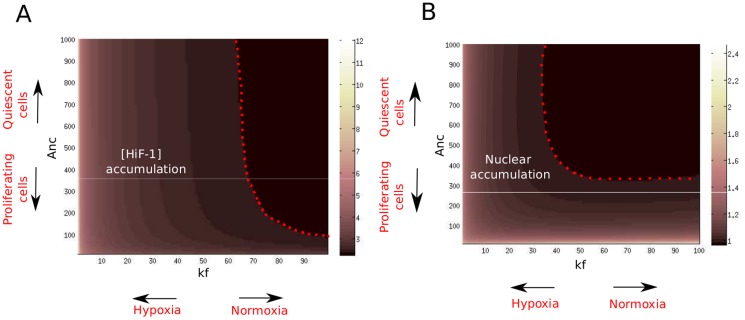
HiF-1*α* accumulation and localization as a function of hypoxia intensity and pVHL export rate. A. The total (nuclear+cytoplasmic) HiF-1*α* equilibrium level, normalized to its value obtained with the default parameters, is plotted as a function of the pVHL-export rate (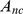) and of the pVHL/HiF-1*α* complex formation rate (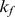). To the left of the red dotted line, total HiF-1*α* significantly increases with respect to the default value. B. Ratio between nuclear and cytoplasmic HiF-1*α* levels as a function of 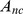 and 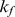. To the left of the red dotted line, HiF-1*α* accumulates in the nucleus.

We then consider a hypoxic event. Hypoxia strongly diminishes the affinity of pVHL for HiF-1*α* [4], [32], [33]. This effect can be simulated by decreasing the value of the complex formation rate (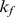). The equilibrium levels of cytoplasmic and nuclear HiF-1*α* were calculated with different values of 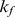, with 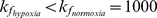, in order to simulate the effects of hypoxia on HiF-1*α* accumulation. Figure 3 shows the nuclear and cytoplasmic level of HiF-1*α* for 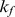 in the range [0.1 100]. We notice that our simulated HiF-1*α* accumulation is very similar to the exponential curve determined by Jiang et al. (1996) [26]. Our model reproduces well the switch-like accumulation of HiF-1*α* when oxygen pressure diminishes. When 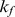 passes a threshold (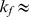 2), HiF-1*α* accumulates in the nucleus. This value is coherent with data from the literature measuring the affinity of pVHL for HiF-1*α* as a function of HiF-1*α* hydroxylation. Illingworth et al. (2010) [32] and Hon et al. (2002) [33] showed that hydroxylation of the ODD-domain multiplied 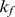 by three orders of magnitude at least. When 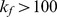, HiF-1*α* is close to its normoxic level (obtained for 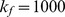), and is present in equal concentration in the nucleus and in the cytoplasm. The value of 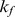 which determines the switch (accumulation of HiF-1*α* in the nucleus) is linked to the nucleo-cytoplasmic export rate (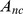) of the pVHL/HiF-1*α* complex. As shown in the literature, this export rate is supposed to vary between cells lines, and notably between proliferating and quiescent cells. As we noticed in the introduction, differentiated and slowly proliferating cells can be characterized by high values of the pVHL-export rate 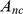, whereas non-differentiated, proliferating cells export pVHL with a lower rate. We aim to study the influence of hypoxia and pVHL export rate (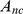) on HiF-1*α* level and localization. We made a map of the total (nuclear and cytoplasmic) HiF-1*α*, normalized to its concentration in normoxia for a non-proliferating cell (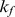 = 1000, 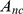 = 1000), as a function of 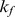 and 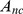 (Figure 4 A). We also represented the ratio between nuclear and cytoplasmic HiF-1*α* concentrations: R = [HiF-1*α*]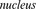/[HiF-1*α*]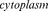 (Figure 4 B). We can notice that when 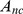 is close to its default value (1000, case of slowly-proliferating cells), HiF-1*α* is homogeneously present in the nucleus and in the cytoplasm during normoxia (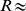
1). When hypoxia is intense (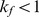), HiF-1*α* accumulates in the nucleus (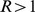). When 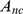 is low (proliferating cells), HiF-1*α* tends to accumulate in the nucleus even in normoxia.

### Simulation of HiF-1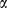 kinetics under hypoxia

**Figure 5:**
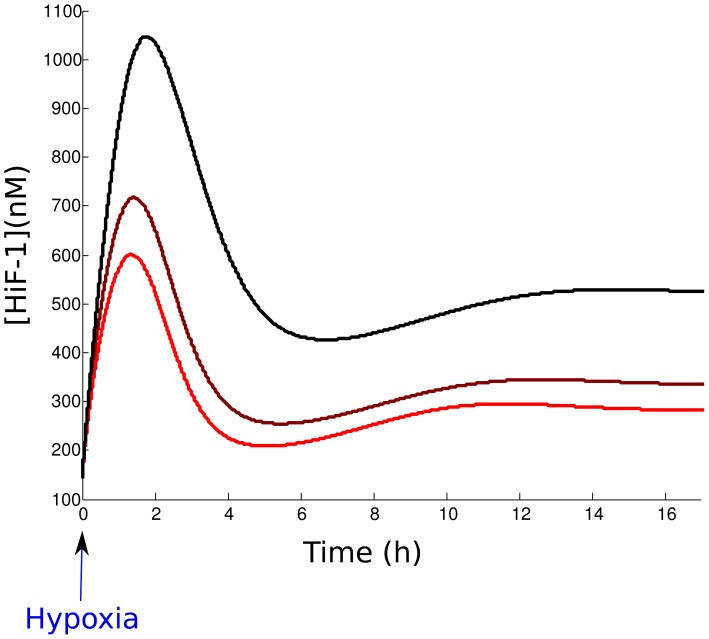
Simulation of the temporal evolution of HiF-1*α* during a hypoxic event. From the red curve to the black curve: simulations for increased levels of hypoxia, with 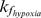 = 10, 5, 1.

We now simulate the kinetics of nuclear HiF-1*α* accumulation under hypoxia. To that end, we first fix the system to its equilibrium state in normoxia, determined by the parameters presented in Table 4. In that case the HiF-1*α* concentration at the equilibrium is 141 nM. Then, we run a simulation with a new value of 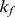, taking the equilibrium state as the initial condition of the system. This protocol simulates a hypoxic event, because we perturb the system at the equilibrium state by choosing hypoxic values of 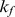, such as 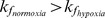. Figure 5 represents the temporal evolution of nuclear HiF-1*α* after the beginning of three hypoxic events of increasing intensities: 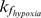 = 10, 5, 1. The evolution of HiF-1*α* concentration is in agreement with data from the literature, with a rapid increase for about two hours up to a maximum value, and a decrease down to an equilibrium value. When it reaches its maximal value, HiF-1*α* concentration is multiplied by 4–8 with respect to its normoxic value, depending on the intensity of hypoxia. This result is coherent with data from the literature [24], [25].

### Simulation of the effects of reoxygenation

**Figure 6:**
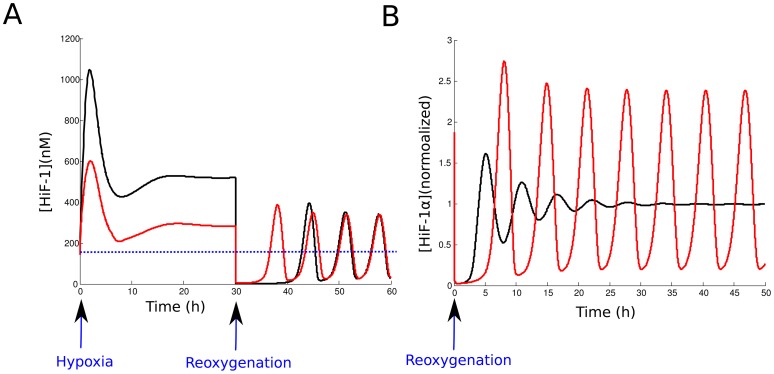
Effect of a hypoxia/reoxygenation sequence on nuclear HiF-1*α* level. A. The level of nuclear HiF-1*α* is plotted during two 30 h hypoxic events of different intensities, followed by reoxygenation. Red: 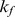 = 10. Black: 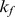 = 1. The dotted blue line corresponds to the HiF-1*α* concentration in normoxia. B. Response to reoxygenation in our compartmentalized model (red curve) and in a non-compartmentalized model of HiF-1*α* regulation (black curve). The nuclear HiF-1*α* was normalized to its value at normoxia

We now test the effect of reoxygenation on HiF-1*α* level. To do so, we follow the same approach than in the simulation of hypoxic events. We calculate the stationary state of the system with a hypoxic value of 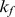 (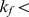
1000). Then, we take this hypoxic stationary state as the initial value of the system, and we run the simulation with the normoxic value of 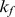 (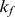 = 1000). This situation simulates a reoxygenation event. We plotted the evolution of nuclear HiF-1*α* level. Figure 6.A shows this temporal evolution during a sequence hypoxia/reoxygenation made for 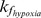 = 1 or 10. We note that reoxygenation leads to a destabilization of HiF-1*α* level, which enters an oscillatory mode with a delay depending on hypoxia intensity. The maximal value reached after reoxygenation by the nuclear concentration of HiF-1*α* is about 2.5-fold its normoxic value. In the case of a moderate hypoxia (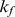 = 10), it is more important than the stationary value in hypoxia. We can notice that the oscillations are exclusively nuclear. The cytoplasmic level of HiF-1*α* remains low after reoxygenation (data not shown). Besides, we could show that this reoxygenation-induced instability of nuclear HiF-1*α* level is linked to the pVHL import/export dynamics. Figure 6.B compares the molecular response to reoxygenation of our compartmentalized model and of a non-compartmentalized model. To consider a non-compartmentalized model, we imposed 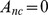 and we plotted the temporal evolution of HiF-1*α*, normalized to the value for normoxia. The instabilities of the nuclear HiF-1*α* level are strongly attenuated in this case. Thus, compartmentalization of HiF-1*α* is crucial to generate reoxygenation-induced instabilities.

### Simulation of HiF-1*α* level in tumor cells

Different types of mutations can affect HiF-1*α* regulation in cancer cells, leading to its pathological accumulation [67]. Genetic deregulation of HiF-1*α* synthesis can promote a 3-8-fold increase of HiF-1*α* mRNA level [17]–[19]. In our model, this corresponds to a 3-8-fold increase of the HiF-1*α* synthesis rate (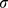). Yoshikawa et al. (2006) [18] observed in peritoneal cancers a 3-fold increase of mRNA and a 2-fold increase in VEGF mRNA expression. As VEGF is a target gene of HiF-1, we can consider that an increase of VEGF expression reflects a similar increase in HiF-1*α* protein level. Second, pVHL activity can be impaired. It is notably the case in Renal Clear Carcinoma (RCC), known to be due to different types of pVHL mutations [15], [16]. In these types of cancer, HiF-1 level is not due to genetic overexpression, since some authors have found a constant level of HiF-1 mRNA [68], [69]. Wiesener et al. (2001) [69] found a 6-7-fold increase of VEGF mRNA in RCC.

**Figure 7:**
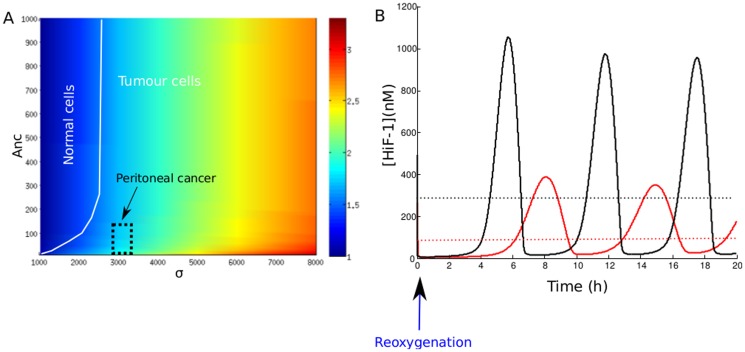
Consequences of a pathological overexpression of HiF-1*α*. A. Accumulation of HiF-1*α* in 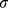 mutated cells, compared to normal cells. The ratio [HiF-1*α*]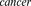/[HiF-1*α*]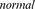 is plotted as a function of 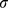 (HiF-1*α* synthesis rate) and 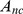 (pVHL export rate). The black dotted square indicates the location of the peritoneal cancer studied by Yoshikawa et al. (2006) [18], characterized by a 2-fold increase of HiF-1*α* protein and a 3-fold increase of HiF-1*α* mRNA. B. Effects of reoxygenation on normal cells, and mutated cells. Reoxygenation after hypoxia is simulated, and the evolutions of nuclear HiF-1*α* concentration are plotted in the case of normal (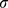 = 1000, red curve), and 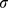 mutated cells (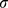 = 5000, black curve). The dotted lines represents the normoxic equilibrium levels in each case.

First, we aim to simulate the effects of a genetic deregulation of HiF-1*α* expression in the normoxic level of the protein. We calculated the stationary value of the total (nuclear and cytoplasmic) HiF-1*α* level as a function of the HiF-1*α* synthesis rate (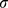), and of the pVHL export rate (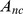). We considered this last parameter because it is supposed to vary with cell state. As explained previously, non-proliferative and differentiated cells are supposed to exhibit a high value for 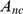, whereas proliferative cells have a low pVHL-export rate. We normalized this value with the level of HiF-1*α* calculated for a normal cell, characterized by the default values of Table 4. Figure 7.A presents the value of this ratio R =  [HiF-1*α*]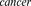/[HiF-1*α*]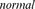 as a function of 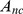 and 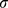. The increase of HiF-1*α* synthesis rate (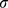) makes HiF-1*α* to accumulate in normoxia compared to the non-mutated case. The calculated increase of HiF-1*α* (multiplied by 1–4) is coherent with data from the literature. We could place on the diagram the position of the peritoneal cancer studied by Yoshikawa et al. (2006) [18]. The space we delimited corresponds to a 2-fold increase of HiF-1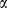 protein and a 3-fold increase of HiF-1*α* mRNA, corresponding to a 3-fold of 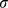. We also notice that low values of the pVHL export rate (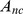) enhances the effect of mutations related to the alteration of the HiF-1*α* production rate (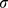).

We then compare the response to a hypoxia/reoxygenation signal of normal cells, and cells presenting a deregulation of HiF-1*α* expression (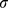 mutated cells). We simulated the effects of a reoxygenation after a hypoxic event, and we plotted (Fig. 7.B) the evolution of total HiF-1*α* for 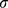 = 1000 (normal cells) and 5000 (tumor cells). We note that the oscillations are amplified in the tumor cells. Indeed, the ratio between the maximal value of the oscillations and the normoxic stationary level of HiF-1*α* is equal to 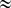 3.2 for tumor cells, and to 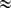 2.5 for normal cells. Thus, our model predicts that mutations of the HiF-1 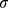 synthesis rate promotes tumorigenesis because it increases the cellular level of HiF-1*α*, and because it amplifies the instabilities due to reoxygenation.

**Figure 8:**
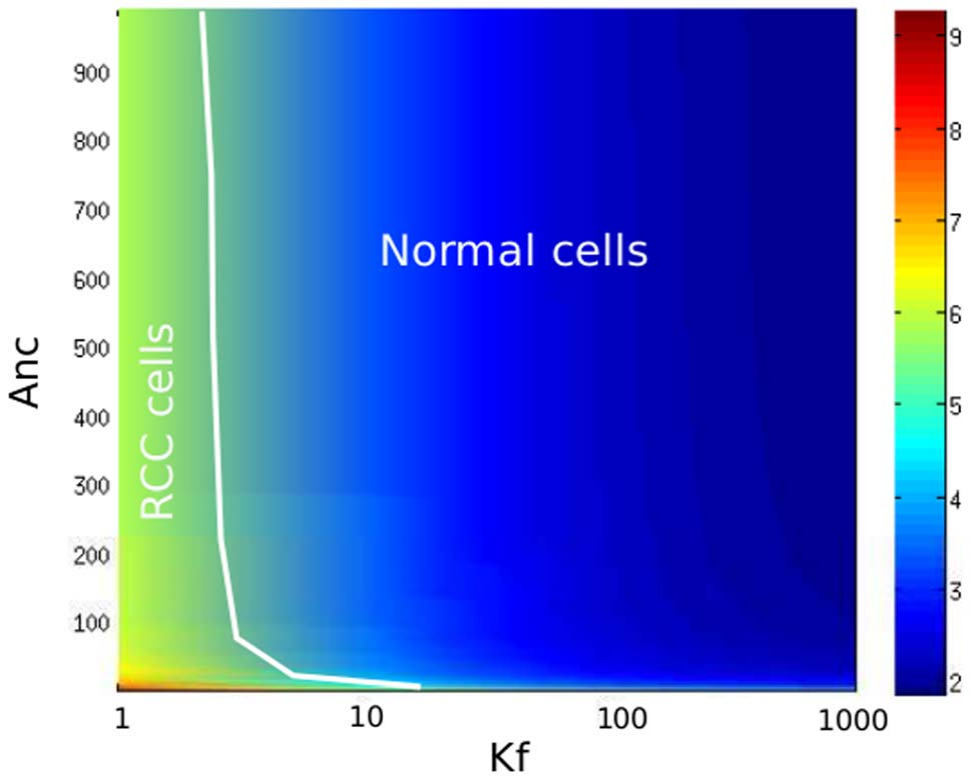
Consequences of pVHL mutations. Accumulation of HiF-1*α* in tumor cells with mutated pVHL. The value of R =  [HiF-1*α*]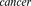/[HiF-1*α*]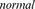 is plotted as a function of 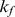 (pVHL/HiF-1*α* complex formation rate) and 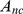 (pVHL export rate). The white line limits the space of the RCC cancer cells studied by Wiesener et al. (2001) [69].

Second, we aim to quantify the effects of pVHL mutations on HiF-1*α* accumulation. We plotted the value of R =  [HiF-1*α*]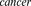/[HiF-1*α*]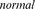 as a function of the pVHL/HiF-1*α* complex formation rate (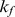), and of the pVHL export rate (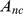). The result was plotted in a (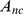, 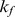) diagram (Figure 8). We can locate on this diagram the RCC cancer cells studied by Wiesener et al. (2001) [69]. These cells present a mutation on pVHL, and a 6-7-fold increase of HiF-1*α* genetic activity. Our model leads to the hypothesis that the pVHL/HiF-1*α* complex formation rate is divided by about 100 in this type of cancer.

### Simulation of the effects of therapies

**Figure 9:**
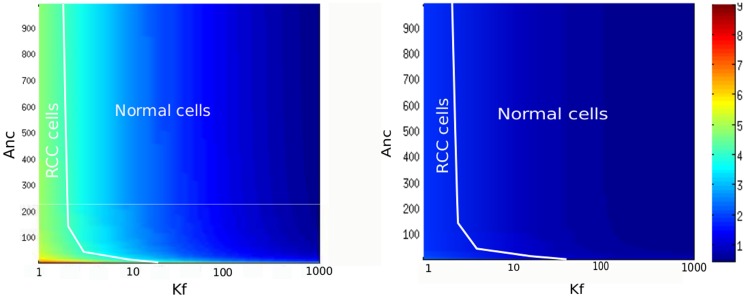
Effects of siRNA therapy in pVHL mutated cells. Nuclear Accumulation of HiF-1*α* in the tumor case, without or with therapy. We calculated the value of 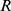 =  ([HiF-1*α*]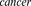/[HiF-1*α*]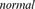) (in the nucleus) as a function of 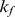 (complexation rate) and 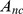 (pVHL export rate). A. Without siRNA therapy (HiF-1*α* synthesis rate 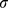 = 1000). B. With siRNA therapy (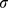 = 200).

**Figure 10:**
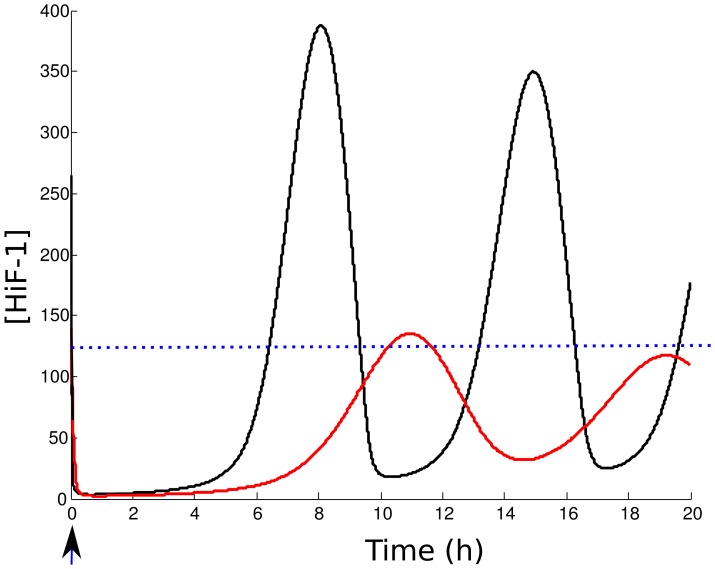
Effects of siRNA therapy on HiF-1*α* level evolution after reoxygenation. Reoxygenation after a hypoxic event is simulated for the HiF-1*α* synthesis rate 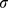 = 1000 (black curve) and 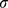 = 200 (red curve). The blue dotted line represents the normoxic level for a normal cell.

Since it is overexpressed in many cancer, HiF-1*α* became a target for anti-cancer therapies [3]. We can use our model to predict the efficiency of different therapeutic strategies. Our model suggests that a therapy which aims to regulate HiF-1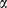 has to reduce its nuclear normoxic level, but also to attenuate the instabilities induced by reoxygenation. A first therapeutic strategy we can test consists in using siRNA to reduce the synthesis of HiF-1*α* [70]. This corresponds to a decrease of the synthesis rate of HiF-1*α* from its default value (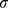 = 1000). We consider a cancer cell presenting mutations of pVHL (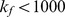), and we calculated the ratio R =  [HiF-1*α*]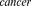/[HiF-1*α*]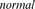 in the nucleus as a function of 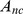 (pVHL export rate) and 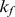 (pVHL/HiF-1*α* complexation rate) for 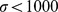. We chose 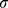 = 200. We consider here the nuclear level because it is responsible for the genetic activity of HiF-1*α*. This decrease of the HiF-1*α* synthesis rate (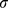) simulates the action of the siRNA. Figure 9 compares the nuclear HiF-1*α* accumulation without therapy and with siRNA therapy. We note that a 5-fold decrease of HiF-1*α* expression is efficient to decrease the HiF-1*α* level. For instance, the RCC cells localized in the diagram, presenting a 6-7-fold increase of nuclear HiF-1*α* level without treatment, and a 2-fold increase after treatment. We then test if the siRNA therapy is able to attenuate the instabilities generated by reoxygenation. Figure 10 shows that a decrease of 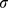 attenuates the oscillations. HiF-1*α* does not exceed any more the normoxic value for a normal cell.

**Figure 11:**
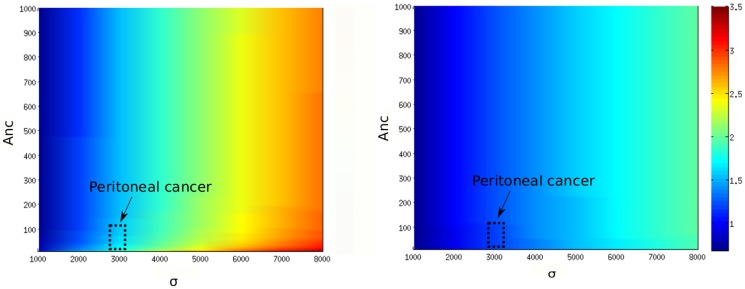
Effects of an increased proteasomal activity on nuclear HiF-1 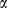 level of 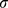-mutated cells. The value of 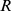 =  [HiF-1*α*]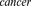/[HiF-1*α*]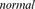 is plotted as a function of 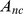 (pVHL export rate) and 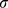 (HiF-1*α* synthesis rate). Left: non-treated cells. Right: result after a 10-fold increase of pVHL-dependent degradation of cytoplasmic and nuclear HiF-1*α* degradation rates (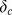 and 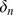).

**Figure 12:**
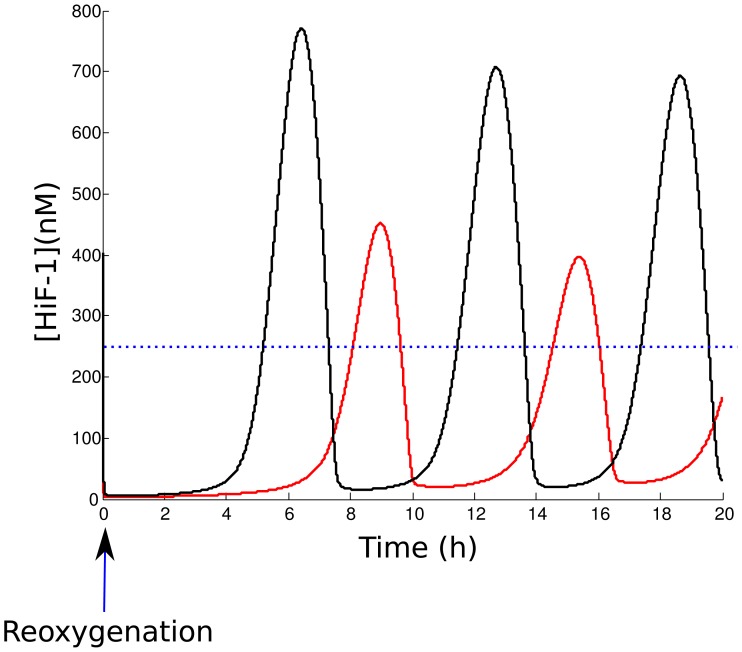
Effects of an increased proteasomal activity on HiF-1*α* evolution after reoxygenation. Reoxygenation after a hypoxic event was simulated for non-treated cells (black curve), and with a 10-fold increase of pVHL-dependent HiF-1*α* degradation rate 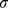 (red curve). The cell considered is a tumor cell, with a deregulation of HiF-1*α* expression (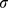 = 3000) The blue dotted line represents the normoxic level for this cell.

Then, we test an other therapeutic strategy, which consists in promoting the pVHL-dependent degradation of HiF-1*α* [71]. We modeled this effect by increasing the pVHL-dependent cytoplasmic and nuclear HiF-1*α* degradation rates, 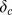 and 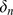. We first compute the effects of this therapy on nuclear HiF-1*α* normoxic level for a tumor cell presenting a deregulation of HiF-1*α* expression (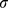-mutated cells). We considered a 10-fold increase of the pVHL-dependent degradation rates (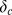 and 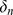). Figure 11 shows the value of 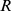 =  [HiF-1*α*]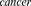/[HiF-1*α*]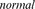 in the nucleus as a function of 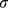 (HiF-1*α* synthesis rate) and 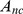 (pVHL export rate). We notice that HiF-1*α* level strongly decreases when the treatment is applied in normoxia. For instance, the peritoneal cancer localized in the diagram presents a 2-fold increase of HiF-1*α* level when untreated, and a 1.2-fold increase after treatment. We then test if this therapy attenuated the instabilities due to reoxygenation. Figure 12 shows the evolution of HiF-1*α* after reoxygenation for a 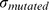 tumor cell (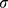 = 3000), with or without treatment. We notice that the oscillations are not significantly attenuated. Even if the pVHL-dependent degradation rate had been multiplied by 10, the amplitude would still exceeds the normoxic level. Thus, our model predicts that this strategy is not efficient to suppress the effects of reoxygenation on HiF-1*α* accumulation.

## Discussion

In this paper, we construct and use a mathematical model of HiF-1*α* regulation. We consider, in agreement with the literature, that the protein is present both in the nucleus and in the cytoplasm. One originality of the model is that the protein is degraded in these two compartments in a pVHL-dependent manner. The constitutive pVHL-shuttle ensures the exchanges of HiF-1*α* between the cytoplasm and the nucleus. We consider a simple regulatory model, directed by the oxygen-dependent affinity of pVHL for HiF-1*α*, and a feedback of HiF-1*α* on pVHL synthesis.

This point of view is original and interesting, because it changes the traditional simulation approach of HiF-1*α* regulation. Indeed, a current field of research tries to find the core pathways of the complete HiF-1*α* regulation network responsible for its observed behavior, notably the switch-like accumulation [60]. To answer this question, the previous models often consider the oxygen-dependent action of PHDs, and some of the numerous feedbacks on the HiF-1*α* regulation system, such as the activation of PHDs synthesis by HiF-1 [56], or the product inhibition by succinate [55]. However, the feedback on pVHL synthesis was never considered. Here, we aim to show that the simple pVHL/HiF-1*α* system, together with a compartmentalization aspect, can explain all essential behavior of HiF-1*α* regulation.

First, we show that our model explains well the dynamics of HiF-1*α* degradation in normoxia, measured by Moroz et al. (2009) [25]. Besides, the dynamics of HiF-1*α* accumulation is simulated in agreement with experimental data. Finally, we could quantify the nuclear and cytoplasmic equilibrium accumulation of the protein under hypoxia. We found a switch-like evolution of the cellular level of HiF-1*α*, with a strong accumulation when the pVHL-HiF-1*α* complex formation rate is decreased by three orders of magnitude, as demonstrated experimentally [32]. Since our model differentiates cytosolic and nuclear HiF-1*α*, we could compare the protein level in these two compartments, as a function of hypoxia and of the pVHL export rate. We found that the accumulation of HiF-1*α* is nuclear in hypoxia, as known in the literature. In normoxia, cytoplasmic and nuclear levels are low and equivalent. Thus, a threshold value of the complexation rate, 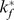, determines the beginning of the nuclear accumulation. When 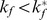, HiF-1*α* accumulates in the nucleus. Our model predicts that the dynamics of the pVHL shuttle is important to determine the value of 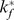. Cells with high pVHL-export rate (corresponding, according to the literature, to slowly proliferating cells) exhibits a lower 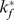 than highly proliferating cells, with a weak pVHL-export rate. This result is consistent with experimental data, showing that quiescent, differentiated cells tend to accumulate HiF-1*α* equally in the cytoplasm and the nucleus, whereas highly proliferating cells exhibit a strong nuclear accumulation [41]. Thus, our model confirms that the difference observed between cells in the pVHL export rate [38] can explain the differences in HiF-1*α* localization. As a consequence, the bi-compartments model, with a cytoplasmic and nuclear degradation, explains in a satisfactory way various experimental data about HiF-1*α* localization, induction, and degradation dynamics.

Second, our model provides interesting results about the molecular responses to reoxygenation. Experimentally, it was observed that reoxygenation causes specific molecular and cellular response. HiF-1*α*-dependent apoptosis observed during reoxygenation, and the enhancement of the synthesis of HiF-1*α*-related genes show that HiF-1*α* activity exhibits an interesting behavior during reoxygenation. The observation of a counter-intuitive increase of HiF-1*α* level by Conde et al. (2012) [47] confirms this idea. Our model is able to generate a specific response to reoxygenation event which is in agreement with these experimental observations.

During a simulated hypoxic event, HiF-1*α* accumulation does not generate instabilities. The protein rapidly reaches an equilibrium level, depending on oxygen pressure in a switch-like manner. On the contrary, brutal reoxygenation generates strong instabilities of the protein level. It decreases suddenly, and after 3 hours, enters into an oscillatory mode, with a large amplitude. The maximal value of HiF-1*α* reached after hypoxia/reoxygenation is about 2.5-fold higher than the normoxic level, and can exceed the hypoxic level. We also observed that this reoxygenation-induced instability was specific to our compartmentalized model. If we do not consider exchanges between cytoplasm and nucleus, the oscillations are strongly attenuated. Thus, the presence of HiF-1*α* instabilities is directly due to the compartmentalization of its degradation dynamics. This oscillatory behavior is biologically relevant. Indeed, it is well known that the oscillatory pattern of transcription factors promotes the expression of specific genes, which are not activated in the case of a stable level of the protein. Thus, reoxygenation-mediated oscillations of HiF-1*α* can explain the synthesis of proteins which are not synthesized during a hypoxic event. Besides, the links existing between HiF-1 and the p53-dependent apoptosis pathways can lead to formulate the following hypothesis [8]. The oscillations of HiF-1*α* generate strong oscillations of p53, which is a known death signal [64]. Thus, it could explain the HiF-1-dependent massive apoptosis observed in some cells after reoxygenation [52]. Besides, our model predicts that these oscillations are exclusively nuclear. The cytoplasmic level remains low after reoxygenation. The biological interest of this behavior is clear. Since HiF-1*α* is a transcription factor, its activity is concentrated in the nucleus. Thus, the biological effects of the oscillations are maximized. Clearly, more experimental works are needed to test if these oscillations are effectively present after a hypoxia/reoxygenation event. The fact that they were not observed until now can be due to a lack of data, or to the fact that other unidentified biological mechanisms attenuate HiF-1*α* instabilities. However, the important conclusion of this paper is that our model of HiF-1*α* regulation, based on experimental data, simulates a specific molecular response to hypoxia/reoxygenation signal. More precisely, we show that our simple HiF-1*α*/pVHL system generates strong instabilities of HiF-1*α* level following reoxygenation. Thus, it gives a possible factor contributing to the similar tendency experimentally observed on HiF-1*α* level and genetic activity.

Finally, we used our model to simulate the effects of known mutations of HiF-1*α* pathways in tumor cells. Notably, we could test the influence of mutations impairing pVHL function, and compare this to a case of Renal Cell Carcinoma studied in the literature [69]. Reciprocally, we used the model to quantify the efficiency of different therapeutic strategies, such as siRNA-mediated inhibition of HiF-1*α* expression, and promotion of its proteasomal degradation. Clearly, as it is an over-simplified model, it cannot reflect the exact response of the HiF-1*α* regulation system to mutations and anti-cancer therapies. However, it provides methods to include these aspects in a ODEs model of HiF-1*α* regulation, in a compartmentalized context. For instance, our model enables us to underline that efficient anti-HiF-1*α* strategies have to decrease its equilibrium level, but also to attenuate the instabilities generated by reoxygenation. In the future, it would be necessary to plug this pVHL/HiF-1*α* model into a more complete system, including the PHDs and all the feedbacks known in literature, to make our test of therapies more useful.

As a conclusion, we can notice that the principal interest of our model is to find a new core-system of the HiF-1*α* regulation network. Our system is based on a oxygen-dependent affinity of pVHL for HiF-1*α*, a feedback of HiF-1*α* on pVHL mRNA synthesis, and the existence of a nucleo-cytoplasmic pVHL shuttle. We show that this simple model, built on the basis of experimental data, explains all the well known essential behaviors of HiF-1*α* regulation. Besides, our model exhibits a specific response to reoxygenation event, which provides a possible factor contributing to experimental observations about HiF-1*α* level and genetic activity during reoxygenation. Finally, our model also provides a method to take into account the HiF-1*α*/pVHL feedback, and the compartmentalization aspect in a future more complete model.

## Supporting Information

Material S1 **Describes the methods used to determine the stationary state of the system, and to simulate hypoxia or reoxygenation events.**
(PDF)

Material S2 **Provides the Matlab code used to obtain the main results of the paper.**
(ZIP)
