# Supplementary material for: Role of Compartmentalization on HiF-1α Degradation Dynamics during Changing Oxygen Conditions: A Computational Approach
Source: PLoS One. 2014 Oct 22;9(10):e110495. doi: 10.1371/journal.pone.0110495 (PMC4206521; doi:10.1371/journal.pone.0110495)
Supplement: Material S1 — Describes the methods used to determine the stationary state of the system, and to simulate hypoxia or reoxygenation events. (PDF) [file pone.0110495.s001.pdf]

Supporting Material S1
Calculation of the stationary state and procedure to run the simulations

All the simulations were performed with Matlab, in the 2012a version.

Stationary state

To compute the value of the stationary concentrations for a given set of parameters, we first reduced the seven equations of the system to the following system:

$$\begin{aligned}
\sigma - \alpha h_n^{eq} - k_f h_n^{eq} v_n^{eq} + kbc_n^{eq} &= 0 \\
v_n^{eq} + c_n^{eq} - B(h_n^{eq})^2 &= 0 \\
v_c^{eq} + c_c^{eq} - C(h_n^{eq})^2 &= 0 \\
c_n^{eq} A - k_f h_n^{eq} v_n^{eq} - A_{cn} c_n^{eq} &= 0 \\
c_c^{eq} A' - k_f h_c^{eq} v_c^{eq} - A_{nc} c_n^{eq} &= 0 \\
h_c^{eq}(k_f v_c^{eq} + \alpha) - k_b c_c^{eq} &= 0
\end{aligned}$$

With:

$$\begin{aligned}
A &= k_b + \delta_n + \gamma + \alpha + A_{nc} \\
A' &= k_b + \delta_c + \gamma + \alpha + A_{cn} \\
B &= \frac{(A_{cn} + \gamma) k_{tl} k_t}{\beta\gamma(\gamma + A_{nc} + A_{cn})} \\
C &= \frac{B A_{nc}}{\gamma + A_{cn}}
\end{aligned}$$

Then, we used the *fsolve* function to find the values of $h_n^{eq}, v_n^{eq}, c_n^{eq}, c_c^{eq}, h_c^{eq}, v_c^{eq}$. The complete version of the program used to compute the values of the variables as a function of the parameters is given in Supplementary Materials B (*equilibrium* and *resol* functions). These function were used to calculate the stationary value of HiF-1α as a function of the k_f parameter (Supplementary Material B , *switch_kf* function). We also used these functions (*equilibrium* and *resol*) to plot the diagrams which give the stationary value of HiF-1α as a function of σ, A_{nc}, k_f.

Simulation of hypoxic and reoxygenation events

To solve the ODEs system, we used the *ode45* function, which gives a numerical solution using a fourth order Runge-Kutta method. The initial conditions given to the solver were always the stationary state of the system, calculated with the method presented previously. We impose to the system a (hypoxic or normoxic) value of k_f to calculate its equilibrium state. Then, the ODEs system is solved with an other value of k_f, to simulate a hypoxic or a reoxygenation event. The full code is available in Supplementary Material (Supplementary Material B, *model* function).
